# Maternal whole blood cell miRNA-340 is elevated in gestational diabetes and inversely regulated by glucose and insulin

**DOI:** 10.1038/s41598-018-19200-9

**Published:** 2018-01-22

**Authors:** Laura Stirm, Peter Huypens, Steffen Sass, Richa Batra, Louise Fritsche, Sara Brucker, Harald Abele, Anita M. Hennige, Fabian Theis, Johannes Beckers, Martin Hrabě de Angelis, Andreas Fritsche, Hans-Ulrich Häring, Harald Staiger

**Affiliations:** 1 Institute for Diabetes Research and Metabolic Diseases of the Helmholtz Zentrum München at the Eberhard Karls University Tübingen, Tübingen, Germany; 2 German Center for Diabetes Research (DZD), Neuherberg, Germany; 3 Institute of Experimental Genetics, Helmholtz Zentrum München, German Research Center for Environmental Health, Neuherberg, Germany; 4 Institute of Computational Biology, Helmholtz Zentrum München, German Research Center for Environmental Health, Neuherberg, Germany; 5 Department of Dermatology and Allergy, Technical University of Munich, Munich, Germany; 6 Department of Obstetrics and Gynaecology, University Hospital Tübingen, Tübingen, Germany; 7 Department of Internal Medicine, Division of Endocrinology, Diabetology, Angiology, Nephrology and Clinical Chemistry, University Hospital Tübingen, Tübingen, Germany; 8 Chair for Experimental Genetics, Technical University München, Neuherberg, Germany; 9 Interfaculty Center for Pharmacogenomics and Pharma Research at the Eberhard Karls University Tübingen, Tübingen, Germany; 10 Institute of Pharmaceutical Sciences, Department of Pharmacy and Biochemistry, Eberhard Karls University Tübingen, Tübingen, Germany

**Keywords:** Epigenetics, Medical research

## Abstract

The number of pregnancies complicated by gestational diabetes (GDM) is increasing worldwide. To identify novel characteristics of GDM, we studied miRNA profiles of maternal and fetal whole blood cells (WBCs) from GDM and normal glucose tolerant (NGT) pregnant women matched for body mass index and maternal age. After adjustment for maternal weight gain and pregnancy week, we identified 29 mature micro-RNAs (miRNAs) up-regulated in GDM, one of which, i.e., miRNA-340, was validated by qPCR. mRNA and protein expression of PAIP1, a miRNA-340 target gene, was found down-regulated in GDM women, accordingly. In lymphocytes derived from the mothers’ blood and treated *in vitro*, insulin increased and glucose reduced miRNA-340 expression. In fetal cord blood samples, no associations of miRNA-340 with maternal GDM were observed. Our results provide evidence for insulin-induced epigenetic, i.e., miRNA-dependent, programming of maternal WBCs in GDM.

## Introduction

The number of pregnancies complicated by gestational diabetes (GDM) is increasing worldwide^1^. Epidemiological studies have indicated that prior occurrence of GDM exerts adverse effects on maternal health by increasing type 2 diabetes (T2D) incidence^1^. However, the underlying mechanisms by which GDM enhances the risk to develop T2D later in life remain elusive. Long-term effects of the maternal lipid metabolism^2^, insufficient compensatory insulin secretion^3^ as well as persisting inflammatory events have been suggested to promote the onset of maternal T2D^4,5^. Besides its detrimental effects on maternal health, GDM has also been shown to impede fetal health^6^ and amplify the offspring’s prevalence to develop metabolic diseases, such as obesity and T2D later in life^7–9^. Epigenetic reprogramming in response to GDM has been suggested to play a major role in defining the future prevalence of T2D in the mother and child. Altered DNA methylation patterns elicited by GDM have been observed in placenta and the offspring’s liver further highlighting the importance of epigenetic reprogramming on the offspring’s long term health outcome^10,11^. Over the past few years several classes of non-coding RNA species, including microRNAs (miRNAs), PIWI-interacting RNAs (piRNAs), small nuclear RNAs (snoRNAs) and long non-coding RNAs (lncRNAs) have emerged as pivotal regulators of the epigenetic landscape with widespread implications on cellular and organismal homeostasis through post-transcriptional gene silencing, transposon silencing, DNA methylation, imprinting, RNA processing and alternative splicing^12–14^. Recent experimental evidence suggests that the expression of these non-coding RNA species changes in response to metabolic and environmental cues and may play a role in the etiology of T2D^15–18^. To see whether these RNA species have a role in GDM, whole blood cell (WBC) protein coding and non-coding transcriptomes were compared between healthy pregnancies and those complicated by GDM. Furthermore, by collecting cord blood from children born from NGT and GDM pregnancies, we studied the potential transmission of the identified maternal transcriptome differences to the fetal blood compartment. Finally, by using freshly isolated lymphocytes we were able to discern which metabolic stimuli can elicit similar transcriptional changes *in vitro*.

## Results

### Patient characteristics

**Figure 1:**
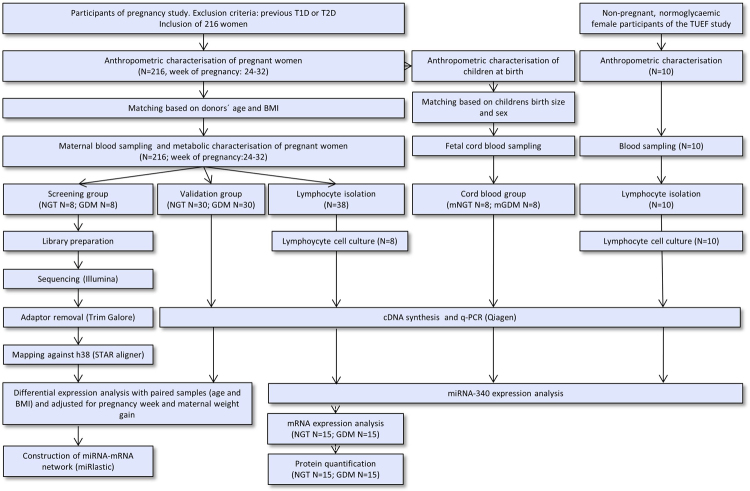
Criteria for participant selection and work flow. Whole blood was collected from eight normal glucose tolerant (NGT) and eight gestational diabetes (GDM) pregnant women for genome-wide transcriptome analysis (screening group). The samples were matched based on age and body mass index (BMI) and were derived from the pregnancy cohort. After whole blood RNA isolation, sequencing (Illumina), library preparation and biostatistical analysis (miRlastic) was conducted. Thirty NGT and thirty GDM women were selected for q-PCR experiments (validation group). Quantification of miRNA-340 only was conducted in lymphocytes collected from pregnant NGT and GDM women (N = 38) (lymphocyte donors). From this group either lymphocyte cell culture or protein quantification was conducted. Additionally, cord blood samples were collected from eight children from NGT pregnancies (mNGT) and eight from GDM pregnancies (mGDM). Finally, cell culture and miRNA-340 quantification was conducted in lymphocytes of non-pregnant controls.

Figure 1 presents the workflow of the study including the criteria for participant selection of the screening, the validation, the lymphocyte donor populations as well as the cord blood population. The anthropometric and metabolic characteristics of the eight Caucasian pregnant NGT and eight pregnant GDM women of the screening population are shown in Table 1. The groups did not significantly differ in maternal age, BMI during pregnancy, family history of parental diabetes as well as time-point of blood sampling and weight gain during pregnancy. Statistical trends (0.06 ≤ P ≤ 0.1) were present in fasted and 2-h glucose levels of the OGTT. Women with GDM had significantly (P ≤ 0.05) increased 1-h glucose and fasting insulin values and decreased insulin sensitivity index (ISI). Similar associations were found in the pregnant women of the validation and lymphocyte donor groups shown in Table 1. Finally, anthropometric characteristics of the children (birth size, birth weight and sex) and of the non-pregnant controls (age, BMI) are also given in Table 1.

**Table 1:** Anthropometric and metabolic characteristics of different participant groups.

	Screening group			Validation group			Cord blood group			Lymphocyte donors			Non-pregnant controls
	NGT	GDM	P	NGT	GDM	P	mNGT	mGDM	P	NGT	GDM	P	NGT
N	8	8	—	30	30	—	8	8	—	20	18	—	10
Age [years]	33 ± 5	32 ± 3	0.7	32 ± 4	31 ± 4	0.4				32 ± 4	31 ± 4	0.3	35 ± 8
Body mass index [kg/m^2^]	28.1 ± 5.4	29.1 ± 6.1	0.7	29.5 ± 5.6	29.8.2 ± 4.07	0.7				27.6 ± 4.0	28.0 ± 4.2	0.2	22.5 ± 3.0
Pregnancy week [weeks]	23.0 ± 9.5	25.9 ± 1.7	0.4	27.6 ± 2.37	27.0 ± 2.3	0.4				26.5 ± 2.0	24.8 ± 7.2	0.3	—
Fasting glucose [mmol/L]	4.44 ± 0.27	4.89 ± 0.62	0.09	4.58 ± 0.3	4.87 ± 0.47	**0.005**				4.43 ± 0.21	4.79 ± 0.51	**0.01**	—
1-h glucose [mmol/L]	7.76 ± 1.21	10.90 ± 0.82	**0.00013**	7.97 ± 1.64	10.41 ± 1.52	**0.0001**				8.17 ± 1.08	10.79 ± 1.01	**0.0003**	—
2-h glucose [mmol/L]	6.32 ± 1.31	7.52 ± 1.44	0.1	6.36 ± 1.27	7.94 ± 1.71	**0.002**				6.49 ± 1.06	7.83 ± 1.5	**0.01**	—
Fasting insulin [pmol/L]	59.88 ± 28.30	126.38 ± 53.70	**0.01**	68.71 ± 28.15	116.76 ± 44.99	**0.006**				68.71 ± 28.15	116.76 ± 45.0	**0.006**	—
Insulin sensitivity index[10^19^ L^2^/mol^2^]	13.6 ± 6.8	5.0 ± 2.0	**0.03**	9.63 ± 4.83	5.46 ± 2.25	**0.00005**				5.54 ± 2.02	7.92 ± 6.43	0.1	—
Maternal weight gain [kg]	6.53 ± 3.38	11.27 ± 9.64	0.2	6.91 ± 3.53	8.52 ± 6.39	0.9				7.37 ± 5.64	7.39 ± 9.64	0.4	
Family history of parental diabetes	Paternal = 0 Maternal = 0	Paternal = 1 Maternal = 0	0.5	Paternal = 0 Maternal = 0	Paternal = 4 Maternal = 1	0.3				Paternal = 0 Maternal = 0	Paternal = 2 Maternal = 1	0.8	
Birth weight [kg]							3.38 ± 0.59	3.36 ± 0.51	0.9				
Birth size [cm]							51.2 ± 1.6	51.0 ± 2. 6	0.8				
Fetal sex [female]							4	4	—				

Table 1 shows anthropometric and metabolic characteristics of the sequencing, validation, cord blood, lymphocyte donor group and non-pregnant control women. All data are given as absolute numbers or means ± SD. Significant differences calculated in a Student’s t-test or Chi-square-test (family history of diabetes) are marked by using bold fonts. Missing values are indicated with a minus. Blank table cells refer to not applicable data.

### miRNA expression patterns in WBCs related with maternal GDM

**Figure 2:**
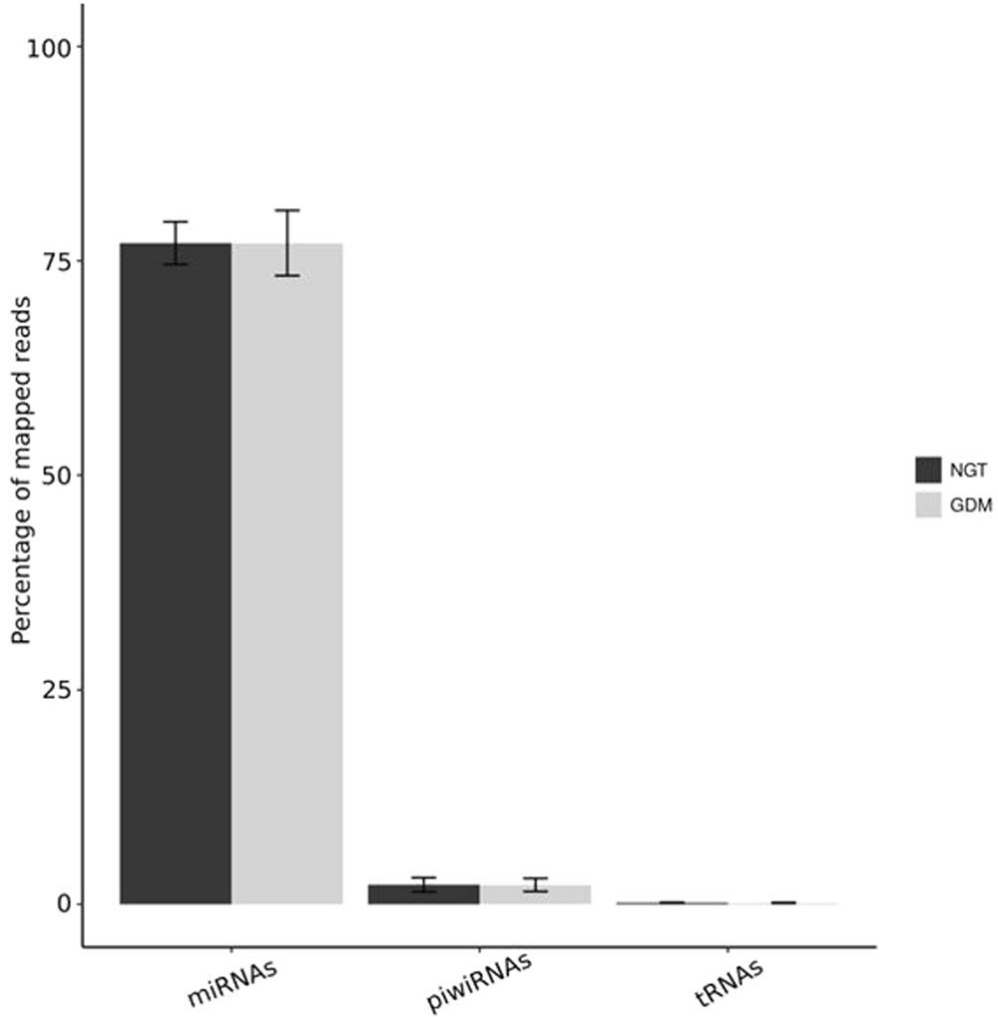
Mapping of reads within sncRNA species to annotated genomic regions. The majority of the reads could be mapped to micro RNAs (miRNA) annotated regions. Minorities of the reads could be associated with reads annotated with piwi-interacting RNAs (piRNA) and transfer RNAs (tRNAs). Reads of sncRNA of normal glucose tolerant (NGT) pregnant women are shown in white bars. Reads of PBMCs of gestational diabetes (GDM) women are shown in black bars.

The main research question of this study was to identify differential RNA expression in WBCs between NGT and GDM women during pregnancy. sncRNA, lncRNA, and mRNA expression analysis was conducted in the screening population. As shown in Fig. 2, the vast majority of the identified unique mappings among the non-coding RNA species are miRNAs (~80%). Less than 5% of the reads belonged to piRNA and tRNA species. This indicates that most of the effects regarding epigenetic reprogramming can be expected in the group of the miRNAs.

**Figure 3:**
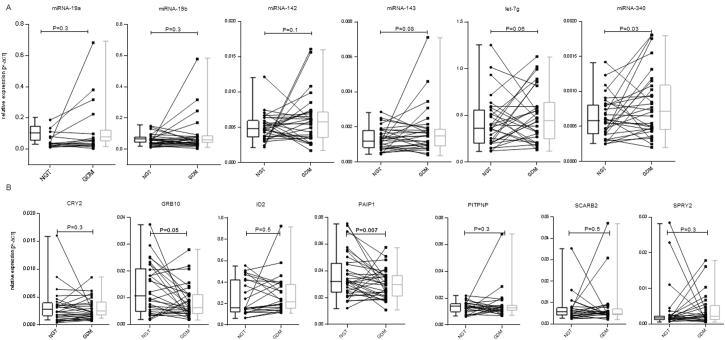
Validation of miRNA and mRNA sequencing results within the validation groupby q-PCR. Sequencing results were validated within the validation group of 30 normal glucose tolerant (NGT) and 30 gestational diabetes (GDM) pregnant women by q-PCR. (**A**) Six miRNAs were selected for q-PCR validation experiments within the validation. (**B**) Seven mRNAs, which are all predicted targets of miRNA-340 were selected for q-PCR validation experiments. Samples were paired based on maternal age and body mass index. Data was adjusted for pregnancy week and weight gain till the end of second trimester. Shown are the means ± SD. Differences considered as statistically significant (P ≤ 0.05) are marked by bold fonts.

In order to investigate GDM-related effects on the miRNAs, a paired analysis was conducted. The participants were matched based on BMI and maternal age, and data was additionally adjusted for gestational weight gain and pregnancy week. As shown in Table 2, 29 significantly increased mature miRNAs were found increased with GDM (P < 0.05; false discovery rate [FDR] < 0.1). No GDM-related reduction in miRNA expression was detected. No significant differences in the expression of piRNAs and tRNAs between both groups were detected. For q-PCR validation experiments, five miRNAs with the highest fold-changes and with interaction sites within mRNAs negatively associated with GDM in the screening population were selected (miRNA-19a, -142, -143, -340, and let-7g). Additionally, miRNA-19b was selected for q-PCR validation, as this miRNA was, based on the network analysis, proposed to be a central miRNA related to GDM (see below). These six miRNAs selected for q-PCR validation are marked in Table 2 by using bold fonts. In order to get independent validation, q-PCR analyses were conducted in WBCs from a substantially larger validation group of 30 NGT and 30 GDM women. Anthropometric and metabolic data of this validation subgroup is shown in the Table 1. As shown in Fig. 3A, miRNA-340 was found significantly elevated in GDM (P = 0.03). For miRNA-142, miRNA-143 and let-7g marginal increased expression related with GDM were present (0.06 ≤ P ≤ 0.1). MiRNA-19a, and −19b were not significantly related with GDM (P ≥ 0.3, both). Expressions relative to paired NGT samples are shown in Supplemental Figure S1A.

**Table 2:** MiRNAs up-regulated in maternal GDM in whole blood cells collected during pregnancy (sorted by fold-changes).

Name	FC	logCPM	P	FDR
miRNA-199a-3p	2.213	3.917	7.50E-03	0.083
miRNA-199b-3p	2.213	3.917	7.50E-03	0.083
miRNA-15a-5p	2.169	9.190	4.88E-03	0.072
**miRNA-19a-3p**	2.154	5.782	1.48E-03	0.035
miRNA-96-5p	2.036	7.029	1.65E-04	0.020
**let-7g-5p**	1.965	12.247	4.32E-04	0.024
**miRNA-143-3p**	1.946	7.242	8.72E-04	0.028
**miRNA-340-5p**	1.907	6.828	7.06E-04	0.028
**miRNA-142-5p**	1.882	11.583	3.36E-04	0.024
miRNA-1307-5p	1.870	5.413	1.04E-03	0.028
let-7f-5p	1.836	11.930	5.32E-03	0.072
miRNA-107	1.825	9.919	6.44E-04	0.028
let-7c-5p	1.749	4.566	1.81E-04	0.020
miRNA-18a-5p	1.735	4.410	8.56E-03	0.088
miRNA-660-5p	1.730	6.842	2.15E-03	0.041
miRNA-17-3p	1.728	5.856	2.15E-04	0.020
**miRNA-19b-3p**	1.629	9.256	5.28E-03	0.072
miRNA-106b-5p	1.617	8.228	5.65E-03	0.073
let-7a-5p	1.611	12.127	1.10E-03	0.028
miRNA-451a	1.606	17.754	3.96E-03	0.067
let-7e-5p	1.589	3.544	8.35E-03	0.088
let-7i-5p	1.589	11.408	6.85E-03	0.082
miRNA-148a-3p	1.584	11.695	4.21E-03	0.067
miRNA-22-3p	1.577	13.539	9.01E-04	0.028
miRNA-17-5p	1.571	9.101	9.47E-03	0.094
miRNA-145-5p	1.569	4.349	6.23E-03	0.078
miRNA-93-5p	1.546	10.233	3.88E-03	0.067
miRNA-103a-3p	1.495	12.179	1.75E-03	0.035
miRNA-103b	1.495	12.179	1.75E-03	0.035

Table 2 lists miRNAs significantly (P < 0.05; FDR < 0.1) associated with GDM in the screening group. The samples were paired based on maternal age and BMI and adjusted for maternal weight gain and pregnancy week. GDM was defined as endpoint variable. Fold-changes (FCs) are shown in the 2^nd^ and logarithmically transformed counts per million (logCPM), indicating the relative abundance of the transcript. are shown in the 3^rd^ column of the table. Uncorrected P-values and false discovery rates (FDRs) are indicated in the 4^th^ and 5^th^ column, respectively. MiRNAs shown in bold fonts indicate those which were selected for q-PCR validation experiments.

### mRNA expression patterns in WBCs related with maternal GDM

In order to investigate associations between mRNA patterns in WBCs and GDM, the mRNA dataset was examined. The same paired analysis approach was made as for sncRNAs. One hundred sixty-three mRNAs were significantly (P < 0.05; FDR < 0.1) reduced in GDM in the screening group and are shown in Supplemental Table S1. For q-PCR validation of differential mRNA expression within the validation group, we specifically selected predicted target mRNAs of miRNA-340 being down-regulated in GDM (P < 0.05; FDR < 0.2) in the screening group (listed in Supplemental Table 2). Different sizes (N = 60 for CRY2, GRB10, PAIP1; N = 48 for ID2, PITPNB, SCARB2, and SPRY3) of the validation groups result from limited sample amount. As shown in Fig. 3B, GDM-related significant reductions in expression could be detected for GBR10 and PAIP1 (P ≤ 0.05, both). For CRY2, ID2, PITPNB, SCARB2, and SPRY3 no significant expression changes in GDM were found. Expressions relative to paired NGT samples are shown in Supplemental Figure S1B.

**Figure 4:**
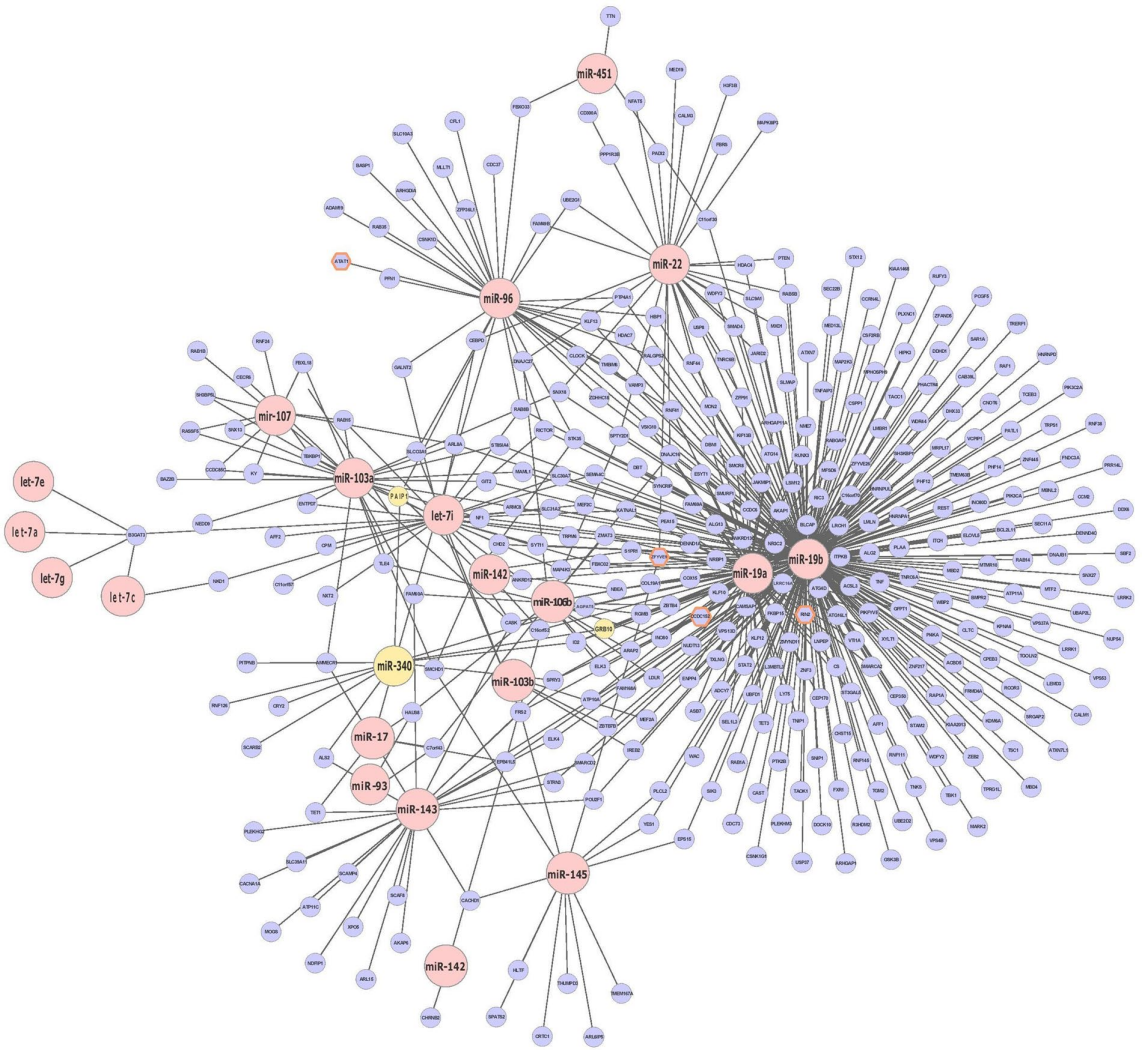
miRNA-mRNA regulatory network inference. MiRNAs positively related with GDM are indicated as red dots (P < 0.05; FDR < 0.1). They are connected with blue dots, indicating predictedtarget mRNAs. Blue dots with an orange frame showed a significant (P < 0.05; FDR < 0.2) down-regulation related with GDM. MiRNA-340, GRB10, and PAIP1 as validated components are marked in yellow. For data analysis the R package miRlastic was used.

The network analysis generated with miRlastic R (Fig. 4) displays the miRNAs significantly elevated in GDM in red (P < 0.05, FDR < 0.1). Predicted target mRNAs of each miRNA are shown in blue. Those mRNAs marked with an orange frame were elevated in GDM in our dataset. MiRNA-340, GRB10 and PAIP1 as validated components of the network are shown in yellow.

### miRNA-340 and GRB10 and PAIP1 mRNA and protein expression in lymphocytes of NGT and GDM women

**Figure 5:**
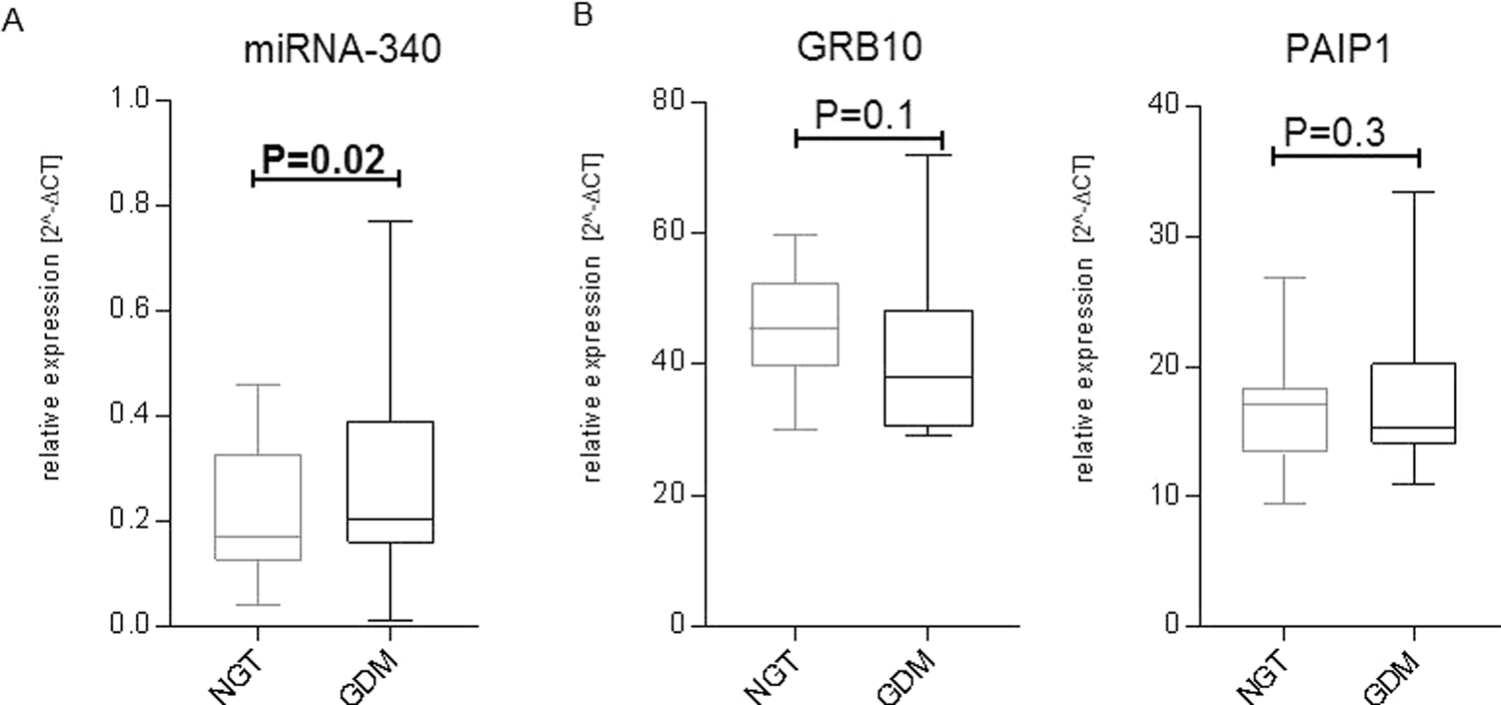
Expression of miRNA-340, GRB10 and PAIP1 in lymphocytes. RNA and proteins from lymphocytes of fifteen NGT and GDM women was collected. Samples were paired based on maternal age and body mass index. Relative expression of miRNA-340 (**A**) and both targets GRB10 and PAIP1 (**B**) was compared. Shown are means ± SD. Statistically significant P-values (P ≤ 0.05) are marked by bold fonts.

To investigate if GDM-related miRNA and mRNA expression changes of WBCs are reflected in of lymphocytes, RNA of this whole blood cell type was collected from fifteen NGT and fifteen age- and BMI-matched GDM women. Next, expression analysis of miRNA-340 and both targets GRB10 and PAIP1 were conducted. As indicated in (Fig. 5A) a significantly elevated expression of miRNA-340 was found in lymphocytes of GDM women (P = 0.02). Only GRB10 mRNA revealed a trend for lower expression in GDM (P = 0.1) (Fig. 5A). Furthermore, we conducted western blots and show the results in Supplemental Figure S2. Protein expression of GRB10 was not different between age and BMI-matched NGT (N = 15) and GDM (N = 15) samples (P = 0.7). In contrast to that, a statistical trend for reduced PAIP1 protein expression was seen in GDM samples after normalization with the reference protein GAPDH (P = 0.06). Regression analyses of miRNA-340 expression with PAIP1 expression was conducted to investigate if miRNA is a relevant regulator of PAIP1 expression. No significant correlation between miRNA-340 and PAIP1 mRNA expression was seen. However, we detected a significant negative correlation of miRNA-340 with PAIP1 protein expression (N = 30; P = 0.006).

### Assessment of miRNA-340 in WBCs from cord blood samples

In order to assess whether maternal GDM–related miRNA expression is reflected in fetal WBCs, cord blood was collected from eight children born from NGT (mNGT) and eight born from GDM (mGDM) pregnancies. The samples were paired based on fetal sex and birth size. Anthropometric data of the children is shown in Table 1. Relative expression value of miRNA-340 was investigated by q-PCR. For miRNA-340, no significant association with maternal GDM was detected, as indicated in Supplemental Figure S3.

### Regulation of miRNA-340 and its target mRNAs by glucose and insulin in human lymphocytes

**Figure 6:**
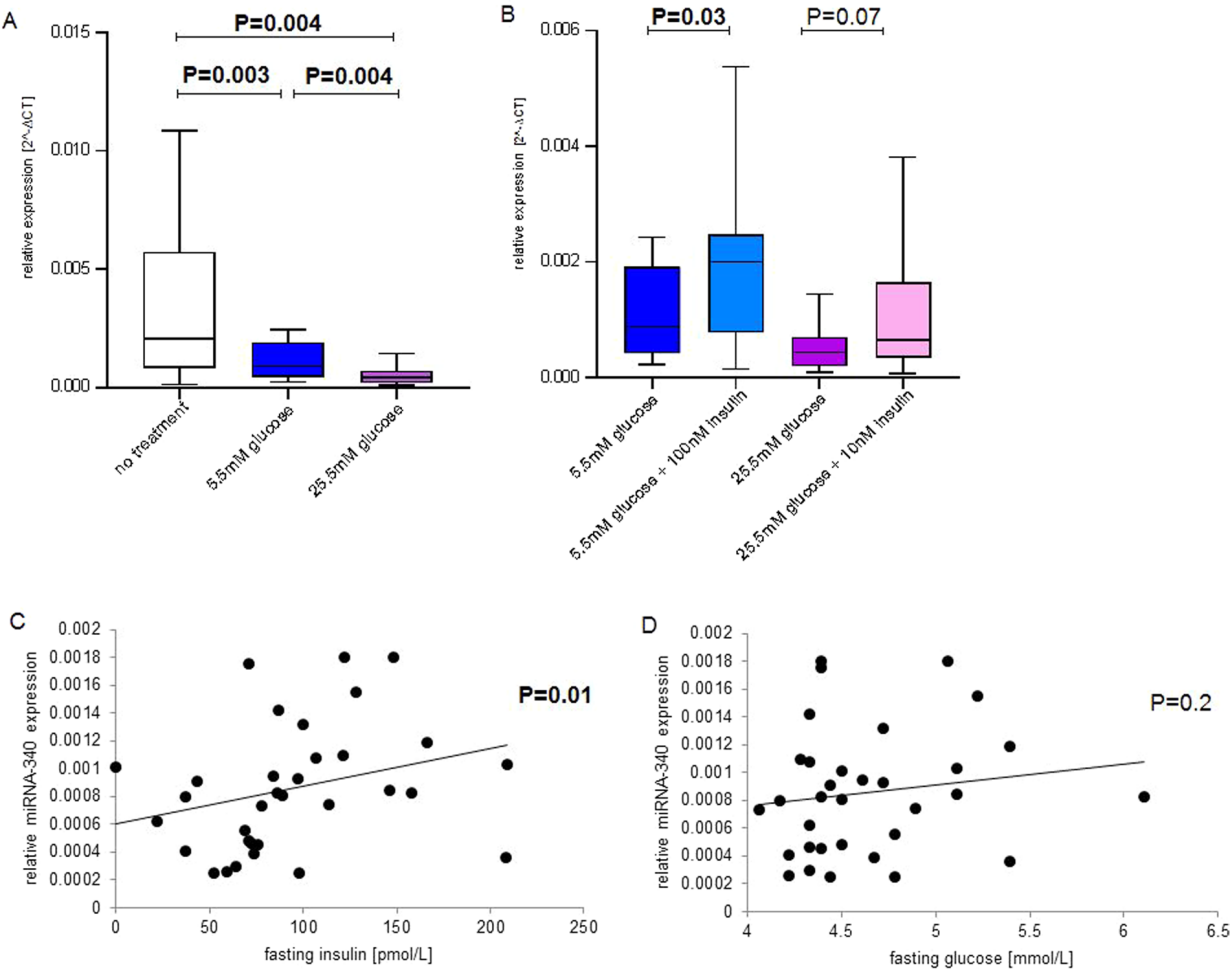
Expression of miRNA-340 in lymphocytes and whole blood cells in response to glucose and insulin miRNA expression in non-cultured and cultured lymphocytes of eight pregnant women was investigated. (**A**) Comparison of non-cultured (no treatment, non-colored bars) and lymphocytes cultured with 5.5 mM (blue bars) or 25.5 mM (violet bars) glucose. (**B**) Comparison of cells cultured in medium containing 5.5 mM glucose without and with 100 nM insulin (light blue bars), respectively, and cells cultured in medium containing 25.5 mM glucose without and with 10 nM insulin (light violet bars), respectively. miRNA expression in whole blood cells (WBCs) in relation to fasting insulin (**C**) and glucose (**D**) levels within the validation cohort (N = 30). Shown are means ± SD. Statistically significant P-values (P ≤ 0.05) are marked by bold fonts.

**Figure 7:**
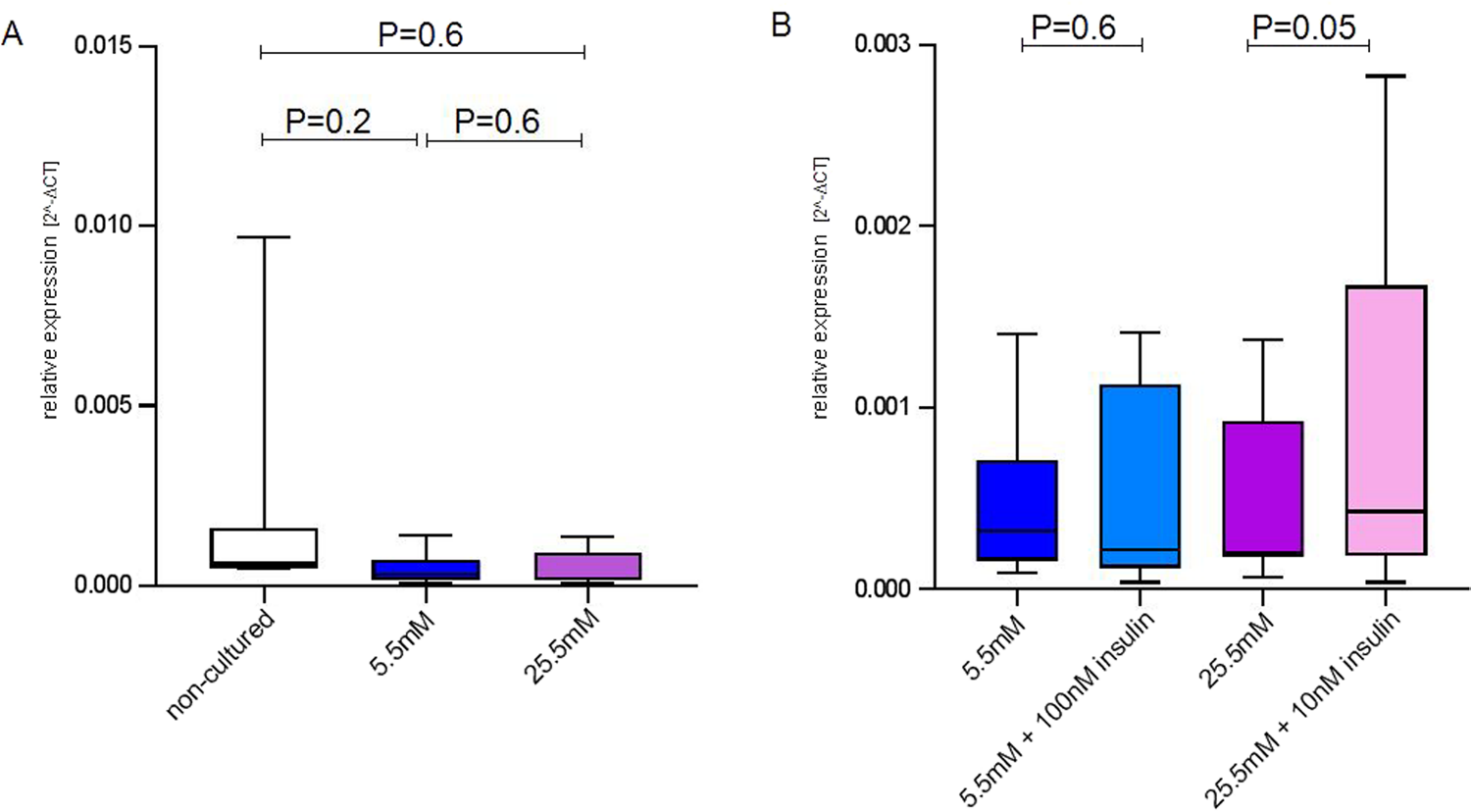
Expression of miRNA-340 in lymphocytes of non-pregnant women stimulated with glucose and insulin. miRNA expression in non-cultured and cultured lymphocytes of eight pregnant women was investigated. (**A**) Comparison of non-cultured (no treatment, non-colored bars) and lymphocytes cultured with 5.5 mM (blue bars) or 25.5 mM (violet bars) glucose. (**B**) Comparison of cells cultured in medium containing 5.5 mM glucose without and with 100 nM insulin (light blue bars), respectively, and cells cultured in medium containing 25.5 mM glucose without and with 10 nM insulin (light violet bars), respectively. Shown are means ± SD. Differences considered as statistically significant (P ≤ 0.05) are marked by bold fonts.

MiRNA-340 was shown to be significantly elevated in GDM by miRNA sequencing (N = 16) in the screening group and by q-PCR analysis in the validation group (N = 60). In order to identify factors responsible for this increased miRNA expression, lymphocytes of eight pregnant women were cultured in medium containing 5.5 mM or 25.5 mM glucose. These two glucose concentrations reflect fasting and postprandial glucose levels, respectively. As shown in Fig. 6A, increased glucose levels were related with significantly reduced (P < 0.05) levels of miRNA-340. Additionally, the effect of insulin at both glucose concentrations was investigated in this experiment. Cells were cultured in medium containing either 100 nM insulin and 5.5 mM glucose or 10 nM insulin and 25.5 mM glucose. The first condition should mimic increased fasting glycaemia and insulin resistance, the second condition impaired insulin secretion in a severely diabetic pregnant woman. Anthropometric and metabolic characteristics of the women donating the lymphocytes are shown in the Table 1. As shown in Fig. 6B, culturing the cells in 5.5 mM glucose and 100 nM insulin led to significantly increased expression value of miRNA-340 (P = 0.03) compared to cells cultured at 5.5 mM glucose without insulin. Stimulation of the cells at higher glucose (25.5 mM) and lower insulin (10 nM) led to a statistical trend (P = 0.07) for increased expression of miRNA-340, compared to stimulation of cells with 25.5 mM glucose. Also, a MANOVA was conducted to investigate differences in lymphocyte glucose and insulin responses between NGT and GDM pregnant women. No interaction effect was present for the miRNA investigated (P ≥ 0.05) (data not shown). This finding indicates that miRNA expression of miRNA-340 responded in a similar manner to different glucose and insulin concentrations in both groups. In order to investigate if *in vitro* effects of insulin and glucose are also present *in vivo*, a linear regression analysis was conducted. Maternal fasting insulin and glucose were correlated with WBC’s miRNA-340, GRB10 and PAIP1 expression. As shown in Fig. 6C, maternal fasting insulin levels were positively associated with WBC miRNA-340 expression (P = 0.01). No significant association was present between miRNA-340 and fasting glucose (Fig. 6D) – even after correction for plasma insulin levels (P = 0.2). Furthermore, no association was seen between fasting insulin and GRB10 or PAIP1 (data not shown). As shown in Fig. 7A and B, lymphocytes of non-pregnant women don’t show the same effects after stimulation with glucose and insulin. Only stimulation with 10 nM insulin at high glucose levels was associated with an increased expression of miRNA-340 (P = 0.05).

### miRNA and mRNA expression patterns in WBCs related with maternal BMI

BMI is an important risk factor for the development of GDM during pregnancy^19^. In order to detect associations between BMI and sncRNA, lncRNA, and mRNA expression, a non-paired secondary analysis was conducted in the screening group. All data was corrected for GDM, maternal age, pregnancy week, and maternal weight gain. In total, four miRNAs showed a positive association with BMI (P < 0.05; FDR < 0.1), as shown in Table 3. Obviously there was no overlap of this set of miRNAs with them found in GDM. The BMI-related differences in miRNA expression were small, as indicated by the low fold-change values. No down-regulated miRNAs were found. Furthermore, no changes in the expression of other analyzed sncRNA species, i.e., piRNAs and tRNAs, were detected.

**Table 3:** MiRNAs related with maternal BMI in whole blood cells collected during pregnancy (sorted by fold-changes)

Name	FC	logCPM	P	FDR
miRNA-4473	1.075	3.575	3.8E-06	0.001
miRNA-199a-5p	1.074	7.601	1.3E-05	0.002
miRNA-339-5p	1.068	7.906	5.8E-05	0.006
miRNA-3653-5p	1.060	6.051	1.3E-03	0.096

Table 3 lists miRNAs positively related with BMI (P < 0.05; FDR < 0.1) within the screening cohort. An unpaired analysis was conducted and adjusted for gestational diabetes, age. maternal weight gain, and pregnancy week. Logarithmic fold changes (FC) are shown in the 2^nd^ and logarithmic counts per million (logCPM), indicating the relative abundance of the transcript, are shown in the 3^rd^ column of the table. Uncorrected P-values and false discovery rates (FDR) as measures of significance of group differences are indicated in the 4th and 5th column.

Additionally, the association of mRNA expression of the WBCs with BMI as an endpoint variable was investigated. As indicated in Supplemental Tables S3, twenty-five mRNAs showed an association with BMI (P < 0.05; FDR < 0.1). In relation to maternal BMI, fifteen mRNAs were down-regulated. Of this group of mRNAs, C6orf10, NCR1, and RMI2 show highest negative fold-changes related with BMI. Furthermore, Supplemental Table S3 shows mRNAs up-regulated with maternal BMI in maternal WBC. Within this group, SLC12A1, coding for a membrane co-transporter and the chemokines CCL3L3 as well as CCL3L1 had most pronounced elevations related with increases in maternal BMI.

## Discussion

miRNAs were shown to have functional relevance in the development of obesity and different types of diabetes^15–17^. Based on these findings, studies have been conducted in order to associate differential miRNA expression patterns with the development, progression, and prevention of diabetes. Additionally to circulating miRNAs^20,21^, transcriptomic changes in blood cells are of growing relevance; as they are easily accessible cells^22–24^.

In order to see if alterations in miRNA-dependent programming related to GDM is detectable in WBCs, we conducted this study. Furthermore, with support of functional studies on human lymphocytes, insights on glucose- and insulin-induced miRNA expression could be collected.

Twenty-nine miRNAs were elevated in GDM within the screening group. Additionally, maternal BMI was selected as endpoint in the genome-wide sequencing analysis. This secondary screening analysis revealed a non-overlapping set of miRNAs to be related with maternal BMI.

To validate the GDM-related RNA-sequencing results, q-PCR experiments were conducted in a larger validation group. Within this validation experiment, miRNA-340 was the only miRNA which could be validated. Significantly elevated miRNA-340 was found in WBC from GDM women. However, miRNA-340 expression was not elevated in all GDM subjects compared to their matched NGT pairs. Further studies are needed to explain these exceptions to evaluate the usability of this miRNA as a possible indicator of GDM. Additionally, quantification of miRNA-340 expression was conducted in isolated lymphocytes. Again, significantly elevated expression was found in GDM women.

Beside of one earlier study describing increased circulating levels of miRNA-340 in newly diagnosed T1D children^25^, miRNA-340 is a newly described miRNA to be differentially expressed in diabetic conditions. As circulating miRNAs were discussed to be derived from blood cells^26–28^, our finding supports this hypothesis. For miRNA-142, miRNA-143, and let-7g statistical trends for elevated expression values in WBCs of GDM-women were found. MiRNA-143 is a miRNA well known to be associated with diabetic conditions: rat peripheral mononuclear blood cells (PBMCs) showed an increased expression of miRNA-143 associated with T2D^29^. Functional relevance of miRNA-143 in diabetes was described and discussed in genetic and diabetic mouse models: an increased expression in murine liver was detected, and miR-143 overexpression was associated with impaired insulin sensitivity and glucose homeostasis^30^. In our screening group, seven members (let-7a, -c, -e, -f, -g, and -i) of the miRNA-let-7 family were highly abundant and positively related with GDM in WBCs in miRNA sequencing. The miRNA-let-7 family was discussed to regulate peripheral glucose metabolism, and modified expression was associated with metabolic diseases^31^. Whole-body transgenic mice overexpressing let-7a, -7d, or 7 f were shown to be glucose-intolerant^32^. In humans, increased expression of let-7a and -7d was shown in skeletal muscle of T2D patients^33^. Furthermore, exposure of HEK293 cells to insulin and glucose resulted in an increased gene expression level of let 7a and -7d^34^. In our miRNA screening group, let-7g had a fold-change of 1.965 with GDM. However, we could not find an elevation in GDM in q-PCR validation experiments.

In order to see how elevated expression of miRNA-340 affects the expression levels of target mRNAs, q-PCR validation of seven target mRNAs was conducted. For GRB10 and PAIP1 significant (P ≤ 0.05) GDM-related reduced mRNA expression was found in maternal WBCs. In maternal lymphocytes of NGT and GDM women, GRB10 and PAIP1 mRNA expression was not significantly different. Furthermore, only a statistical trend for reduced PAIP1 protein expression in lymphocytes of GDM women was observed. However using regression analysis, miRNA-340 negatively correlated with PAIP1 protein expression. Altogether, this indicates that miRNA-340 contributes to the regulation of PAIP1 expression.

In order to investigate functional relations between GDM and the increased expression of miRNA-340, primary cell culture experiments were conducted within our study. A significant insulin effect on the expression of miRNA-340 was observed. Higher glucose levels led to a reduced expression in the presence and absence of insulin. Furthermore, by comparing miRNA expression levels of cultured lymphocytes of NGT and GDM pregnant women no differences in glucose- and insulin-induced changes of miRNA expression were detected. These *in vitro* findings are reflected by a positive correlation of fasting insulin levels with miRNA-340 expression *in vivo* suggesting that GDM-related miRNA derangement in WBCs could result from increased plasma insulin concentrations. Metabolic characteristics indicate that GDM women have significantly increased fasting glucose, 1-h and 2-h insulin levels in the presence of insulin resistance (as indicated by significantly reduced insulin sensitivity values). Similar stimulation experiments were conducted in lymphocytes from non-diabetic, non-pregnant women. Here, no marked expression changes upon insulin or glucose treatment could be observed. These findings suggest that the metabolic and/or hormonal milieu of pregnancy contributes to miRNA expression.

In order to assess whether the maternal WBC miRNA signature can be found in cord blood cells, expression of miRNA-340 in fetal WBCs was quantified. The absence of statistically significant (P ≤ 0.05) alterations in fetal WBCs miRNA expression could be due to the small sample size of the group or the intense clinical management of GDM pregnancies. Increased insulin and glucose levels in cord blood samples of children born from GDM pregnancies were reported^2,35^. The effect of fatty acids on miRNA expression was not investigated in the current study. In future studies, this should be considered. Additionally, the putative reversibility of miRNA derangement should be assessed in GDM follow-up investigations. In summary, our results provide evidence of miRNA-dependent programming of WBCs related with GDM. Functional studies indicate that the GDM-related miRNA-340 responds to insulin and glucose in cultured lymphocytes and could therefore be of importance in hyperinsulinemia-induced changes in gene expression. PAIP1, a predicted target mRNA of miRNA-340, shows reduced mRNA expression related with maternal GDM.

In conclusion, we identified a novel maternal blood-derived miRNA, miRNA-340, that is associated with GDM and induced by insulin in WBC. Our results provide evidence for insulin-induced epigenetic, i.e., miRNA-dependent, programming of maternal WBCs in GDM.

## Material and Methods

### Participants and maternal and fetal blood collection

From a pregnancy study, eight pregnant NGT and eight GDM women, matched for age and BMI, were selected as screening group. For validation, a group of 30 NGT and 30 GDM women from the pregnancy cohort was selected. The anthropometric characterization of the Caucasian women (height, weight, and age) was followed by venous blood collection in pregnancy week 24–32. Additionally, from the Tuebingen Family Study (TUEF) study, blood was collected from 10 healthy non-pregnant women for lymphocyte isolation. The TUEF study is an ongoing life style intervention study in healthy subjects with increased risk for Type 2 diabetes^36^. All measurements and blood collection was conducted after having obtained informed written consent of the participants. Fetal blood was collected from umbilical cord immediately after birth from eight children each from maternal NGT (mNGT) and maternal GDM pregnancies (mGDM). The pregnancy study and TUEF study were approved by the Ethics Committee of the Medical Faculty of the Eberhard Karls University Tuebingen and are therefore performed in accordance with the ethical standards laid down in the Helsinki Declaration.

### Oral glucose tolerance test and clinical chemical analyses

The participants underwent a 5-point 75-g oral glucose tolerance test (OGTT) after overnight fasting. Venous blood samples were obtained at 0, 30, 60, 90 and 120 min for determination of plasma glucose and insulin levels. Blood glucose was determined using a bedside glucose analyzer (YSI, Yellow Springs, OH, USA). Plasma insulin was determined on an ADVIA Centaur XP (Siemens Healthcare GmbH, Erlangen, Germany). NGT was defined as fasting glucose ≤5.11 mmol/L, 1-h glucose ≤10.00 mmol/L; and 2-h glucose ≤8.50 mmol/L. GDM was diagnosed according to the International Association of the Diabetes and Pregnancy Study Groups (IADPSG) recommendations for the diagnosis and classification of hyperglycemia in pregnancy published in 2010 ^37^. Whole-body insulin sensitivity index (ISI) was calculated from glucose and insulin values during the 5-point OGTT as proposed by Matsuda and DeFronzo^38^: 10,000/[c(Glc_0_)*c(Ins_0_)*c(Glc_mean_)*c(Ins_mean_)]^½^(with c = concentration, Glc = glucose and Ins = insulin).

### RNA isolation, library preparation and RNA sequencing

Whole blood was collected in PAXgene Blood RNA Tubes (PreanalytiX) after overnight fasting. Next, total RNA was isolated using the Paxgene Blood miRNA kit (PreanalytiX) according to the manufacturer’s specifications. The quality of the obtained RNA was subsequently evaluated on the Agilent 2100 Bioanalyzer System and samples with a RIN value ≥ 8.0 were used for downstream library construction. NEBNext small RNA library Prep Set for Illumina and NuGen Ovation RNA-Seq system v2 were used according to the manufacturer’s recommendations to generate small and long RNA-Seq libraries. For long RNA-Seq, the obtained cDNA’s were sheared with a Covaris Focused-ultrasonicator prior to adaptor ligation. Finally, libraries were sequenced on an Illumina HiSeq. 2500 platform.

### Cell culture experiments, RNA isolation

Human lymphocytes were isolated from heparinized venous blood samples during pregnancy week 24–32. Briefly, the heparinized blood was diluted with PBS (Lonza) in the ratio 1:1 and layered on Biocoll Separating Solution® (Biochrom). Afterwards, the sample was subjected to centrifugation (500 g for 20 min). The white layer representing the lymphocytes was aspirated gently and transferred aseptically to new tubes. The cell suspension was washed and cultured in sterile DMEM medium (GIBCO) with different glucose (5.5 mM or 25 mM) and insulin (Insuman Rapid, Sanofi) (10 nM or 100 nM) concentrations. The medium was supplemented with 10% fetal bovine serum (Biochrom) and 1% penicillin-streptomycin (Lonza). The number of cells was adjusted to 0.5 × 10^6^ cells/well in 12-well plates. After 20-h incubation at 37 °C, the cells were harvested. Total RNA was isolated with miRNeasy micro kit (Qiagen) from freshly isolated and 20-h stimulated cells.

### cDNA synthesis and quantitative PCR

Reverse transcription was conducted as recommended by the manufacturer. Briefly, 500 ng total RNA was reversely transcribed with 5 × miScript HiFlex Buffer (miScript ll RT kit, Qiagen) under following conditions: 37 °C for 60 min, 95 °C for 15 min, immediately on ice. This was followed by quantitative polymerase chain reaction (q-PCR) (miScript SYBR Green PCR kit, Qiagen). Q-PCR measurements of both miRNAs and mRNAs were conducted in duplicates. For miRNA q-PCR, 10 ng cDNA were used. Relative expression of miRNA-19a-3p (MIMAT0000073: 5′-UGUGCAAAUCUAUGCAAAACUGA-3′), miRNA-19b-3p (MIMAT0000074: 5′-UGUGCAAAUCCAUGCAAAACUGA-3′), miRNA-142-5p (MIMAT0000433: 5′CAUAAAGUAGAAAGCACUACU-3′), miRNA-143-3p (MIMAT0000435: 5′UGAGAUGAAGCACUGUAGCUC-3′), let-7g-5p (MIMAT0000414: 5′UGAGGUAGUAGUUUGUACAGUU-3′), miRNA-340-5p (MIMAT0000750: 5′UCCGUCUCAGUUACUUUAUAGC-3′), and the house keeping gene U6 small nuclear 6, (RNU6B) was determined on a LightCycler 450 (Roche) in following conditions: activation 95 °C for 15 min, quantification (45 cycles) 94 °C for 15 sec, 55 °C for 30 sec, 70 °C for 30 sec. In order to control for primer specificity, a melting curve was performed. For mRNA q-PCR, 2.5 ng of cDNA was used. Relative expression of CRY2 (up 5′-CAAGTCCTTCAGTGGGGA AC-3′, down 5′-CAAGTCCTTCAGTGGGGAAC -3′), GRB10 (up 5′-TTCTGGTAAAGGAGC ATTCCA-3′, down 5′-AGGACGAGCAAACCAGGAC-3′), ID2 (up 5′-CTGGACTCGC ATCCCACTAT-3′, down 5′-TAACTCAGAAGGGAATTCAGAAGC-3′), PAIP1 (up 5′-AATCC TCACAACCATCCTCATAG-3′, down 5′-GAACTCGGAGTCAGCAATGG-3), PITPNB (up 5′-CGAGACTCAGAAAGAACTAGAAACAA-3′, down 5′-TGACCCTACAGGGGACTCAT-3′), SCARB (up 5′-GGCCGATGCTGCTTCTAC-3′, down 5′-CAAATGCCTCAGTACCATTCC-3′), SPRY3 (up 5′-AAATCATCTGTTAGCCCCTAACTC-3′, down 5′-TCATATGAAATGTATCAA GGAACCA-3′), and the house keeping gene RPS13 (up 5′-CCCCACTTGGTTGAAGTTGA-3′, down 5′-ACACCATGTGAATCTCTCAGGA-3′) was determined on the Light Cycler 450 (Roche) as shown earlier^39^.

### Protein detection

Primary lymphocytes were isolated as described above and washed in PBS (Lonza, Basel, Switzerland). Protein isolation was conducted with AllPrep DNA/RNA/Protein Mini Kit (Qiagen, Hilden, Germany) and protein was solved in Lämmli buffer containing 62.5 mM Tris, 2% SDS, 10% Glycin, and 3% beta-mercaptoethanol. Protein concentrations were determined with Qubit Protein Assay Kit (Thermo Fisher Scientific, Dreieich, Germany). The proteins from cell lysates (50 μg) were resolved on a 7.5% Tris-HCl gel and blotted onto nitrocellulose membranes. Unspecific binding sites were blocked with 5% milk TBS-Tween prior to overnight incubation with primary antibodies against GRB10 and PAIP1 both (Abcam, Cambridge, UK, both) (1:1000 in 5% BSA in TBS-Tween, both). For normalization of the protein content, an antibody against GAPDH (Sigmaaldrich, St. Louis, USA) (1:1000 in 5% BSA in TBS-Tween) was used. The incubation with primary antibody was followed by 1 h incubation with a HRP-coupled secondary antibody (1:3000 in 5% milk in TBS-Tween). Protein band detection and quantification was conducted with Image lab software (Biorad, Hercules, USA).

### Biostatistical analysis

For RNA sequencing, raw read counts for miRNAs and mRNAs were generated by overlapping the positions of the mapped reads with annotations obtained from miRbase (doi:10.1093/nar/gkt1114) and RefSeq, (doi:10.1093/bioinformatics/btp616) respectively. Statistical analysis of the read counts can be found at the NCBI Gene Expression Omnibus (GEO record: GSE92772) and were conducted using the edgeR (doi:10.1093/bioinformatics/btp616) package for R. For the identification of GDM-related differential expression of sncRNAs, lncRNAs, and mRNAs, in the screening group and validation group, a paired analysis (two-tailed Student′s t-test) was conducted after adjustment for pregnancy week and maternal weight gain within the first half of the pregnancy. For this paired analysis, P-values < 0.05 and false FDR < 0.1 were considered as significant. For visualization of the results of the screening group, an interaction network analysis was performed. Based on miRNA-mRNA *in silico* target site predictions obtained from Target Scan (version 6.2) (doi:10.1016/j.cell.2004.12.035), the previously described miRlastic R approach for the identification of potential regulatory interactions between significantly regulated miRNAs and mRNAs by incorporating their expression profiles was applied^40^. Briefly, elastic net regression was used for performing feature selection on a set sequence-based target predictions to identify inversely regulated miRNA-target relationships. For q-PCR validation experiments within the validation and cord blood group, paired two-tailed Student’s t-tests were conducted (JMP, SAS, Cary, USA). In cases where the direction of expected changes was defined by earlier experiments one-tailed paired Student’s t-tests were conducted (JMP, SAS, Cary, USA). Using linear regression analysis, WBC miRNA-340 expression was correlated with maternal fasting glucose and insulin levels. Applying multiple regression lymphocyte miRNA-340 expression was correlated with PAIP1 mRNA and protein expression after correction for two batches of sample preparation (JMP, SAS, Cary, USA). In order to investigate effects of maternal BMI, an unpaired analysis of the results from the screening group was conducted, by multiple linear regression analysis (standard least squares method). Adjustments for this secondary analysis were maternal GDM, maternal age, weight gain, and pregnancy week.

### Accession codes

Sequencing data can be found at the Gene Expression Omnibus database (www.ncbi.nlm.nih.gov/geo/) under following GEO record: “GSE92772 - RNA sequencing data of whole blood cells of normal glucose tolerant (NGT) and gestational diabetes (GDM) pregnant women”.

## Electronic supplementary material

Supplemental Figures and Tables
