# Author Correction: Maternal whole blood cell miRNA-340 is elevated in gestational diabetes and inversely regulated by glucose and insulin

**DOI:** 10.1038/s41598-022-11273-x

**Published:** 2022-04-26

**Authors:** Laura Stirm, Peter Huypens, Steffen Sass, Richa Batra, Louise Fritsche, Sara Brucker, Harald Abele, Anita M. Hennige, Fabian Theis, Johannes Beckers, Martin Hrabě de Angelis, Andreas Fritsche, Hans-Ulrich Häring, Harald Staiger

**Affiliations:** 1 Institute for Diabetes Research and Metabolic Diseases of the Helmholtz Zentrum München at the Eberhard Karls University Tübingen, Tübingen, Germany; 2 German Center for Diabetes Research (DZD), Neuherberg, Germany; 3 Institute of Experimental Genetics, Helmholtz Zentrum München, German Research Center for Environmental Health, Neuherberg, Germany; 4 Institute of Computational Biology, Helmholtz Zentrum München, German Research Center for Environmental Health, Neuherberg, Germany; 5 Department of Dermatology and Allergy, Technical University of Munich, Munich, Germany; 6 Department of Obstetrics and Gynaecology, University Hospital Tübingen, Tübingen, Germany; 7 Department of Internal Medicine, Division of Endocrinology, Diabetology, Angiology, Nephrology and Clinical Chemistry, University Hospital Tübingen, Tübingen, Germany; 8 Chair for Experimental Genetics, Technical University München, Neuherberg, Germany; 9 Interfaculty Center for Pharmacogenomics and Pharma Research at the Eberhard Karls University Tübingen, Tübingen, Germany; 10 Institute of Pharmaceutical Sciences, Department of Pharmacy and Biochemistry, Eberhard Karls University Tübingen, Tübingen, Germany

Correction to: *Scientific Reports* 10.1038/s41598-018-19200-9, published online 22 January 2018

This Article contains an error in Table 1 where the mean value and standard deviation of pregnancy week for the "screening group:NGT women" was incorrectly given as 23.0 +/- 9.5.

The correct numbers are 26.5 +/- 2.1.

Incorrect:

**Table:** 

	Screening group		
	NGT	GDM	P
N	8	8	–
Age [years]	33 ± 5	32 ± 3	0.7
Body mass index [kg/m^2^]	28.1 ± 5.4	29.1 ± 6.1	0.7
Pregnancy week [weeks]	23.0 +/− 9.5	25.9 ± 1.7	0.4

Correct:

**Table:** 

	Screening group		
	NGT	GDM	P
N	8	8	–
Age [years]	33 ± 5	32 ± 3	0.7
Body mass index [kg/m^2^]	28.1 ± 5.4	29.1 ± 6.1	0.7
Pregnancy week [weeks]	26.5 +/− 2.1	25.9 ± 1.7	0.5

Furthermore, this results in a correct p value of 0.5 instead of 0.4.

Data described and shown in other Tables and Figures are not affected, as the analyses were performed from separate files.

