# Supplementary material for: Maternal whole blood cell miRNA-340 is elevated in gestational diabetes and inversely regulated by glucose and insulin
Source: Sci Rep. 2018 Jan 22;8:1366. doi: 10.1038/s41598-018-19200-9 (PMC5778051; doi:10.1038/s41598-018-19200-9)
Supplement: Supplementary file 1 — Supplemental Figures and Tables [file 41598_2018_19200_MOESM1_ESM.doc]

#### Maternal whole blood cell miRNA-340 is elevated in gestational diabetes and inversely regulated by glucose and insulin

Laura Stirm^1,2^*, Peter Huypens^2,3^*, Steffen Sass^2, 4^*,Richa Batra^4,5^ Louise Fritsche^1,2^, Sara Brucker^6^, Harald Abele^6^, Anita M Hennige ^1,7^, Fabian Theis^2,4^, Johannes Beckers^2,3,8^, Martin Hrabě de Angelis^2,3,8^, Andreas Fritsche^1,2,7^, Hans-Ulrich Häring^1,2,7,9^, Harald Staiger^1,2,3,9,10^

^*^contributed equally

^1^Institute for Diabetes Research and Metabolic Diseases of the Helmholtz Zentrum München at the Eberhard Karls University Tübingen, Tübingen, Germany

^2^German Center for Diabetes Research (DZD), Neuherberg, Germany

^3^Institute of Experimental Genetics, Helmholtz Zentrum München, German Research Center for Environmental Health, Neuherberg, Germany

^4^Institute of Computational Biology**,** Helmholtz Zentrum München, German Research Center for Environmental Health, Neuherberg, Germany

^5^Department of Dermatology and Allergy, Technical University of Munich

^6^Department of Obstetrics and Gynaecology, University Hospital Tübingen, Tübingen, Germany

^7^Department of Internal Medicine, Division of Endocrinology, Diabetology, Angiology, Nephrology and Clinical Chemistry, University Hospital Tübingen, Tübingen, Germany

^8^Chair for Experimental Genetics, Technical University München, Neuherberg, Germany

^9^Interfaculty Center for Pharmacogenomics and Pharma Research at the Eberhard Karls University Tübingen, Tübingen, Germany

^10^Institute of Pharmaceutical Sciences, Department of Pharmacy and Biochemistry, Eberhard Karls University Tübingen, Tübingen, Germany

Corresponding author: Harald Staiger

Email: [harald.staiger@med.uni-tuebingen.de](mailto:harald.staiger@med.uni-tuebingen.de)

Adress: Institute for Diabetes Research and Metabolic Diseases

of the Helmholtz Center Munich at the University of Tübingen

Otfried-Müller-Straße 10, D-72076 Tübingen, Germany

Phone +49-7071-29-85774

Fax +49-7071-29-5646

Supplemental Figure

A

miRNA-19b

miRNA-19a

miRNA-142

miRNA-143

let 7g

miRNA-340

[2^-∆ CT]

P=0.06

P=0.06

**P=0.04**

**P=0.03**

**P=0.01**

**P=0.006**

B

[2^-∆ CT]

CRY2

GRB10

ID2

PAIP1

PITPNP

SCARB2

SPRY3

P=0.1

P=0.5

P=0.09

P=0.4

P=0.3

P=0.2

**P=0.04**

**Supplemental Figure 1: Validation of miRNA sequencing results within the validation subgroup by q-PCR.**

Six miRNAs (A) and seven mRNAs (B) were selected for q-PCR validation experiments within a validation subgroup of 30 normal glucose tolerant (NGT) and 30 gestational diabetes (GDM) women. Samples were paired based on maternal age and body mass index. The results are shown relative to the paired NGT sample Data was adjusted for pregnancy week and weight gain till the end of second trimester. Shown are the means ± SD. Differences considered as statistically significant (P<0.05) are marked by bold fonts.

NGT

GDM

NGT

GDM

NGT

GDM

NGT

GDM

NGT

GDM

NGT

GDM

A

GDM

GDM

GDM

GDM

GDM

GDM

GDM

GDM

GDM

NGT

NGT

NGT

NGT

NGT

NGT

NGT

NGT

NGT


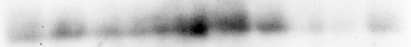

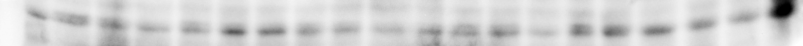

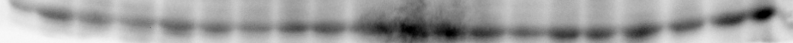


GRB10 (76kDa)

Artificial overlapping spot not considered for quantification


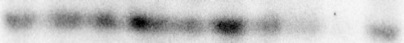


PAIP1 (54 kDa)


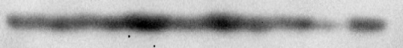

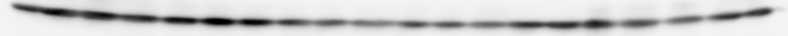


GAPDH (37kDA)


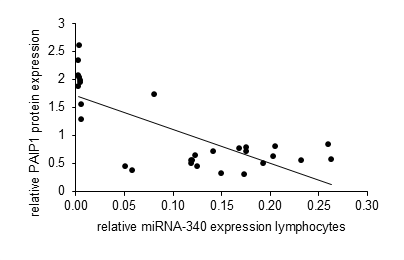

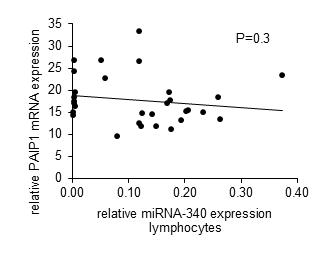


P=0.06

B

C

P=0.7

**P=0.006**

**Supplemental Figure 2: Protein expression of GRB10 and PAIP1 in lymphocytes**

For protein quantification, protein was isolated from lymphocytes of 15 normal glucose tolerant (NGT) and 15 gestational diabetes (GDM) women. Samples were paired based on maternal age and body mass index. A: Western blots with antibodies against GRB10 and PAIP and the reference gene GAPDH. B: Relative protein expression of GRB10 and PAIP1 after normalization with GAPDH. Shown are the means ± SD. C: Correlation of miRNA-340 expression and PAIP1 mRNA and protein expression. Significant correlations after adjustment for batch correction are indicated with bold p-values (P<0.05).

[2^-∆ CT]

miRNA-340

P=0.2

**Supplemental Figure 3: Expression of miRNA-340 in fetal cord blood WBCs.**

miRNA-340 expression in cord blood WBCs of children each born from maternal NGT (mNGT) and maternal GDM (mGDM) (N=8, each) pregnancies was investigated. Samples were paired based on birth size and fetal sex. Shown are the means ± SD.

Supplemental Tables

Supplemental Table 1: MRNAs associated with maternal GDM in whole blood cells collected during pregnancy (sorted by fold-changes)

| Symbol | Name | FC | logCPM | P | FDR |
| --- | --- | --- | --- | --- | --- |
| SFMBT2 | Scm-like with four mbt domains 2 | 0.507 | 3.309 | 1.22E-03 | 0.095 |
| FAIM3 | Fas apoptotic inhibitory molecule 3 | 0.505 | 5.313 | 7.18E-04 | 0.072 |
| LOC100272216 | uncharacterized LOC100272216 | 0.500 | 5.703 | 1.06E-03 | 0.091 |
| CTSW | cathepsin W | 0.494 | 3.278 | 9.91E-04 | 0.090 |
| LILRB1 | leukocyte immunoglobulin-like receptor, subfamily B (with TM and ITIM domains), member 1 | 0.491 | 5.151 | 3.21E-04 | 0.057 |
| CD163 | CD163 molecule | 0.491 | 2.798 | 1.31E-03 | 0.098 |
| ZXDB | zinc finger, X-linked, duplicated B | 0.481 | 2.388 | 1.15E-03 | 0.093 |
| LOC441081 | POM121 membrane glycoprotein (rat) pseudogene | 0.479 | 7.214 | 3.96E-04 | 0.062 |
| ZMAT1 | zinc finger, matrin-type 1 | 0.478 | 2.947 | 6.28E-04 | 0.070 |
| NLRP2 | NLR family, pyrin domain containing 2 | 0.476 | 3.708 | 5.77E-04 | 0.070 |
| LOC100288778 | WAS protein family homolog 1 pseudogene | 0.475 | 3.146 | 6.15E-04 | 0.070 |
| HLA-DMB | major histocompatibility complex, class II, DM beta | 0.473 | 4.998 | 2.55E-04 | 0.052 |
| TAS2R40 | taste receptor, type 2, member 40 | 0.473 | 2.630 | 1.07E-03 | 0.091 |
| NUTM2G | NUT family member 2G | 0.467 | 2.241 | 1.05E-03 | 0.091 |
| TTI1 | TELO2 interacting protein 1 | 0.466 | 2.294 | 1.00E-03 | 0.090 |
| TERF1 | telomeric repeat binding factor (NIMA-interacting) 1 | 0.464 | 2.368 | 7.99E-04 | 0.077 |
| HLA-DMA | major histocompatibility complex, class II, DM alpha | 0.462 | 5.675 | 1.27E-04 | 0.049 |
| ATAT1 | alpha tubulin acetyltransferase 1 | 0.455 | 3.310 | 2.12E-04 | 0.052 |
| MOV10 | Mov10 RISC complex RNA helicase | 0.454 | 1.977 | 1.12E-03 | 0.093 |
| ABCG1 | ATP-binding cassette, sub-family G (WHITE), member 1 | 0.453 | 1.887 | 1.22E-03 | 0.095 |
| ZBTB25 | zinc finger and BTB domain containing 25 | 0.450 | 3.026 | 2.78E-04 | 0.052 |
| GPRASP1 | G protein-coupled receptor associated sorting protein 1 | 0.449 | 1.931 | 1.03E-03 | 0.091 |
| ADGRL1 | adhesion G protein-coupled receptor L1 | 0.448 | 2.225 | 5.59E-04 | 0.069 |
| PRPF31 | pre-mRNA processing factor 31 | 0.446 | 3.825 | 1.36E-04 | 0.049 |
| TSPAN3 | tetraspanin 3 | 0.446 | 2.770 | 2.36E-04 | 0.052 |
| VSTM1 | V-set and transmembrane domain containing 1 | 0.446 | 4.128 | 1.62E-04 | 0.052 |
| TNFSF12 | tumor necrosis factor (ligand) superfamily, member 12 | 0.446 | 1.957 | 6.85E-04 | 0.071 |
| RUNDC1 | RUN domain containing 1 | 0.445 | 2.148 | 5.48E-04 | 0.069 |
| TTBK2 | tau tubulin kinase 2 | 0.444 | 1.971 | 6.64E-04 | 0.071 |
| SFXN3 | sideroflexin 3 | 0.443 | 1.589 | 1.29E-03 | 0.098 |
| LMO7 | LIM domain 7 | 0.443 | 2.073 | 4.83E-04 | 0.066 |
| IGLL5 | immunoglobulin lambda-like polypeptide 5 | 0.441 | 3.061 | 3.72E-04 | 0.059 |
| TYW1 | tRNA-yW synthesizing protein 1 homolog (S, cerevisiae) | 0.440 | 2.195 | 4.61E-04 | 0.065 |
| TXNDC5 | thioredoxin domain containing 5 (endoplasmic reticulum) | 0.438 | 2.072 | 6.50E-04 | 0.071 |
| KIF5C | kinesin family member 5C | 0.438 | 1.973 | 5.53E-04 | 0.069 |
| ARL17A | ADP-ribosylation factor-like 17A | 0.438 | 7.587 | 3.00E-05 | 0.024 |
| WDR46 | WD repeat domain 46 | 0.433 | 3.313 | 1.22E-04 | 0.049 |
| FER | fer (fps/fes related) tyrosine kinase | 0.432 | 1.709 | 1.07E-03 | 0.091 |
| CD40LG | CD40 ligand | 0.431 | 2.076 | 4.94E-04 | 0.066 |
| LHX4-AS1 | LHX4 antisense RNA 1 | 0.429 | 2.390 | 2.09E-04 | 0.052 |
| NAAA | N-acylethanolamine acid amidase | 0.424 | 3.089 | 8.08E-05 | 0.041 |
| GEMIN5 | gem (nuclear organelle) associated protein 5 | 0.424 | 1.764 | 6.21E-04 | 0.070 |
| RHOF | ras homolog family member F (in filopodia) | 0.423 | 1.482 | 1.26E-03 | 0.097 |
| SH3PXD2A | SH3 and PX domains 2A | 0.419 | 2.019 | 4.66E-04 | 0.065 |
| MTHFD1 | methylenetetrahydrofolate dehydrogenase (NADP+ dependent) 1, methenyltetrahydrofolate cyclohydrolase, formyltetrahydrofolate synthetase | 0.419 | 1.612 | 5.67E-04 | 0.069 |
| ZBTB22 | zinc finger and BTB domain containing 22 | 0.414 | 2.056 | 2.58E-04 | 0.052 |
| ST3GAL4-AS1 | ST3GAL4 antisense RNA 1 (head to head) | 0.414 | 2.015 | 5.36E-04 | 0.069 |
| TRMT44 | tRNA methyltransferase 44 homolog (S, cerevisiae) | 0.414 | 1.197 | 1.14E-03 | 0.093 |
| RING1 | ring finger protein 1 | 0.413 | 3.144 | 4.42E-05 | 0.029 |
| GTF2E1 | general transcription factor IIE, polypeptide 1, alpha 56kDa | 0.411 | 1.083 | 1.14E-03 | 0.093 |
| RDH13 | retinol dehydrogenase 13 (all-trans/9-cis) | 0.410 | 4.235 | 3.31E-05 | 0.024 |
| ZNF765 | zinc finger protein 765 | 0.409 | 1.016 | 1.20E-03 | 0.095 |
| TUBGCP5 | tubulin, gamma complex associated protein 5 | 0.406 | 2.407 | 8.88E-05 | 0.043 |
| RHD | Rh blood group, D antigen | 0.403 | 2.908 | 2.16E-04 | 0.052 |
| PRR29 | proline rich 29 | 0.403 | 1.702 | 4.88E-04 | 0.066 |
| ARL17B | ADP-ribosylation factor-like 17B | 0.402 | 4.903 | 7.52E-06 | 0.009 |
| SERF1B | small EDRK-rich factor 1B (centromeric) | 0.400 | 2.490 | 1.88E-04 | 0.052 |
| SERF1A | small EDRK-rich factor 1A (telomeric) | 0.400 | 2.490 | 1.88E-04 | 0.052 |
| ZNF772 | zinc finger protein 772 | 0.397 | 1.076 | 8.39E-04 | 0.080 |
| DDX56 | DEAD (Asp-Glu-Ala-Asp) box helicase 56 | 0.397 | 1.736 | 2.25E-04 | 0.052 |
| RFX5 | regulatory factor X, 5 (influences HLA class II expression) | 0.396 | 1.944 | 1.26E-04 | 0.049 |
| CCDC77 | coiled-coil domain containing 77 | 0.396 | 1.047 | 1.17E-03 | 0.094 |
| TTC27 | tetratricopeptide repeat domain 27 | 0.395 | 1.591 | 2.77E-04 | 0.052 |
| MED22 | mediator complex subunit 22 | 0.395 | 1.404 | 7.10E-04 | 0.072 |
| GLMP | glycosylated lysosomal membrane protein | 0.395 | 0.933 | 1.37E-03 | 0.099 |
| PAAF1 | proteasomal ATPase-associated factor 1 | 0.394 | 0.690 | 1.37E-03 | 0.099 |
| RASGRP3 | RAS guanyl releasing protein 3 (calcium and DAG-regulated) | 0.393 | 1.974 | 2.11E-04 | 0.052 |
| LOC100505549 | uncharacterized LOC100505549 | 0.392 | 1.567 | 2.44E-04 | 0.052 |
| KAT2A | K(lysine) acetyltransferase 2A | 0.390 | 0.695 | 1.32E-03 | 0.098 |
| MPI | mannose phosphate isomerase | 0.385 | 1.383 | 5.94E-04 | 0.070 |
| SNAPC5 | small nuclear RNA activating complex, polypeptide 5, 19kDa | 0.385 | 1.038 | 6.58E-04 | 0.071 |
| LOC100133315 | transient receptor potential cation channel, subfamily C, member 2-like | 0.384 | 0.825 | 9.48E-04 | 0.087 |
| CCHCR1 | coiled-coil alpha-helical rod protein 1 | 0.381 | 1.425 | 4.49E-04 | 0.065 |
| CXXC5 | CXXC finger protein 5 | 0.380 | 1.305 | 2.82E-04 | 0.052 |
| ENTPD6 | ectonucleoside triphosphate diphosphohydrolase 6 (putative) | 0.380 | 1.173 | 5.38E-04 | 0.069 |
| USP21 | ubiquitin specific peptidase 21 | 0.379 | 1.280 | 3.50E-04 | 0.058 |
| KRBA2 | KRAB-A domain containing 2 | 0.376 | 0.925 | 7.42E-04 | 0.074 |
| MLYCD | malonyl-CoA decarboxylase | 0.375 | 0.567 | 1.03E-03 | 0.091 |
| MYO1E | myosin IE | 0.375 | 1.104 | 7.19E-04 | 0.072 |
| NR1D1 | nuclear receptor subfamily 1, group D, member 1 | 0.373 | 1.215 | 3.12E-04 | 0.056 |
| NCR1 | natural cytotoxicity triggering receptor 1 | 0.371 | 1.268 | 7.15E-04 | 0.072 |
| RIN2 | Ras and Rab interactor 2 | 0.369 | 1.058 | 3.74E-04 | 0.059 |
| SLC4A10 | solute carrier family 4, sodium bicarbonate transporter, member 10 | 0.368 | 1.681 | 1.39E-04 | 0.049 |
| LILRA4 | leukocyte immunoglobulin-like receptor, subfamily A (with TM domain), member 4 | 0.368 | 1.416 | 6.49E-04 | 0.071 |
| SNAPIN | SNAP-associated protein | 0.365 | 1.040 | 6.54E-04 | 0.071 |
| SERGEF | secretion regulating guanine nucleotide exchange factor | 0.365 | 0.694 | 7.86E-04 | 0.077 |
| CD72 | CD72 molecule | 0.362 | 0.830 | 6.04E-04 | 0.070 |
| ZNF544 | zinc finger protein 544 | 0.362 | 0.774 | 6.26E-04 | 0.070 |
| PRIM1 | primase, DNA, polypeptide 1 (49kDa) | 0.362 | 1.246 | 4.10E-04 | 0.062 |
| LCMT2 | leucine carboxyl methyltransferase 2 | 0.359 | 0.167 | 1.39E-03 | 0.099 |
| PCDH12 | protocadherin 12 | 0.358 | 0.651 | 1.25E-03 | 0.096 |
| CENPO | centromere protein O | 0.358 | 0.729 | 6.84E-04 | 0.071 |
| DEXI | Dexi homolog (mouse) | 0.355 | 0.500 | 1.20E-03 | 0.095 |
| LOC101927354 | uncharacterized LOC101927354 | 0.354 | -0.514 | 1.36E-03 | 0.099 |
| ZNF304 | zinc finger protein 304 | 0.353 | 0.359 | 1.13E-03 | 0.093 |
| TRIM35 | tripartite motif containing 35 | 0.352 | 1.792 | 5.03E-05 | 0.029 |
| IL21R-AS1 | IL21R antisense RNA 1 | 0.351 | 1.149 | 2.03E-04 | 0.052 |
| CD79A | CD79a molecule, immunoglobulin-associated alpha | 0.350 | 1.188 | 2.80E-04 | 0.052 |
| IGSF8 | immunoglobulin superfamily, member 8 | 0.349 | -0.032 | 1.28E-03 | 0.098 |
| ERI2 | ERI1 exoribonuclease family member 2 | 0.349 | -0.104 | 1.04E-03 | 0.091 |
| DNAJC17 | DnaJ (Hsp40) homolog, subfamily C, member 17 | 0.344 | -0.237 | 1.18E-03 | 0.095 |
| KCNQ5 | potassium channel, voltage gated KQT-like subfamily Q, member 5 | 0.343 | 0.334 | 1.30E-03 | 0.098 |
| NUDCD1 | NudC domain containing 1 | 0.341 | 0.457 | 4.61E-04 | 0.065 |
| FAM226A | family with sequence similarity 226, member A (non-protein coding) | 0.337 | 1.046 | 2.13E-04 | 0.052 |
| FAM226B | family with sequence similarity 226, member B (non-protein coding) | 0.337 | 1.046 | 2.13E-04 | 0.052 |
| DDX49 | DEAD (Asp-Glu-Ala-Asp) box polypeptide 49 | 0.330 | 0.199 | 5.12E-04 | 0.067 |
| PPIAL4D | peptidylprolyl isomerase A (cyclophilin A)-like 4D | 0.330 | 0.806 | 1.69E-04 | 0.052 |
| PPIAL4F | peptidylprolyl isomerase A (cyclophilin A)-like 4F | 0.330 | 0.806 | 1.69E-04 | 0.052 |
| PPIAL4E | peptidylprolyl isomerase A (cyclophilin A)-like 4E | 0.330 | 0.806 | 1.69E-04 | 0.052 |
| NDUFB7 | NADH dehydrogenase (ubiquinone) 1 beta subcomplex, 7, 18kDa | 0.330 | -0.480 | 1.39E-03 | 0.099 |
| ZNF583 | zinc finger protein 583 | 0.330 | 0.047 | 6.19E-04 | 0.070 |
| KLF16 | Kruppel-like factor 16 | 0.328 | -0.117 | 1.32E-03 | 0.098 |
| ZNF540 | zinc finger protein 540 | 0.328 | -0.347 | 1.01E-03 | 0.090 |
| CD83 | CD83 molecule | 0.325 | -0.294 | 1.36E-03 | 0.099 |
| LOC727751 | golgin A2 pseudogene | 0.323 | -0.112 | 8.80E-04 | 0.083 |
| ELAC1 | elaC ribonuclease Z 1 | 0.320 | -0.427 | 1.12E-03 | 0.093 |
| LOC100507639 | uncharacterized LOC100507639 | 0.319 | 1.914 | 2.11E-05 | 0.021 |
| SFXN2 | sideroflexin 2 | 0.319 | -0.043 | 9.41E-04 | 0.087 |
| CCDC152 | coiled-coil domain containing 152 | 0.318 | -0.181 | 7.98E-04 | 0.077 |
| C5orf34 | chromosome 5 open reading frame 34 | 0.317 | -0.495 | 6.85E-04 | 0.071 |
| KIAA0895L | KIAA0895-like | 0.316 | 1.159 | 3.37E-05 | 0.024 |
| LINC01336 | long intergenic non-protein coding RNA 1336 | 0.312 | -0.300 | 5.43E-04 | 0.069 |
| ZFYVE9 | zinc finger, FYVE domain containing 9 | 0.312 | 0.063 | 6.32E-04 | 0.070 |
| BLOC1S4 | biogenesis of lysosomal organelles complex-1, subunit 4, cappuccino | 0.310 | -0.154 | 9.20E-04 | 0.085 |
| PPIAL4C | peptidylprolyl isomerase A (cyclophilin A)-like 4C | 0.310 | -0.254 | 3.48E-04 | 0.058 |
| MPDU1 | mannose-P-dolichol utilization defect 1 | 0.310 | 0.372 | 7.86E-04 | 0.077 |
| DDX51 | DEAD (Asp-Glu-Ala-Asp) box polypeptide 51 | 0.305 | 0.616 | 1.01E-04 | 0.045 |
| RBAK-RBAKDN | RBAK-RBAKDN readthrough | 0.305 | -0.369 | 3.36E-04 | 0.058 |
| DDX12P | DEAD/H (Asp-Glu-Ala-Asp/His) box polypeptide 12, pseudogene | 0.301 | -0.057 | 5.05E-04 | 0.067 |
| TUBA8 | tubulin, alpha 8 | 0.299 | -0.670 | 3.71E-04 | 0.059 |
| SSSCA1 | Sjogren syndrome/scleroderma autoantigen 1 | 0.298 | -0.540 | 4.39E-04 | 0.065 |
| UBE2E2 | ubiquitin-conjugating enzyme E2E 2 | 0.297 | -0.156 | 2.85E-04 | 0.052 |
| FAM223A | family with sequence similarity 223, member A (non-protein coding) | 0.295 | 0.705 | 2.56E-04 | 0.052 |
| FAM223B | family with sequence similarity 223, member B (non-protein coding) | 0.295 | 0.705 | 2.56E-04 | 0.052 |
| SNHG21 | small nucleolar RNA host gene 21 | 0.293 | -0.778 | 3.41E-04 | 0.058 |
| MUC20 | mucin 20, cell surface associated | 0.292 | 2.497 | 2.81E-07 | 0.001 |
| C2 | complement component 2 | 0.290 | 1.360 | 2.34E-04 | 0.052 |
| ZNF256 | zinc finger protein 256 | 0.288 | -0.297 | 4.10E-04 | 0.062 |
| AMER1 | APC membrane recruitment protein 1 | 0.287 | 0.778 | 3.47E-05 | 0.024 |
| C3orf35 | chromosome 3 open reading frame 35 | 0.287 | -0.566 | 4.03E-04 | 0.062 |
| LOC102723927 | uncharacterized LOC102723927 | 0.286 | 1.720 | 1.58E-06 | 0.004 |
| SLC25A23 | solute carrier family 25 (mitochondrial carrier; phosphate carrier), member 23 | 0.284 | 0.342 | 1.39E-04 | 0.049 |
| OR2T3 | olfactory receptor, family 2, subfamily T, member 3 | 0.284 | -0.812 | 2.87E-04 | 0.052 |
| RPS14P3 | ribosomal protein S14 pseudogene 3 | 0.281 | -0.270 | 1.50E-04 | 0.052 |
| MGC70870 | C-terminal binding protein 2 pseudogene | 0.281 | -0.546 | 3.63E-04 | 0.059 |
| OVCH1 | ovochymase 1 | 0.278 | -0.523 | 9.04E-04 | 0.085 |
| SSSCA1-AS1 | SSSCA1 antisense RNA 1 (head to head) | 0.275 | -0.223 | 2.32E-04 | 0.052 |
| SLC35F2 | solute carrier family 35, member F2 | 0.270 | -0.895 | 1.33E-04 | 0.049 |
| STX8 | syntaxin 8 | 0.262 | -0.512 | 1.99E-04 | 0.052 |
| PAX8-AS1 | PAX8 antisense RNA 1 | 0.259 | 0.761 | 9.30E-06 | 0.010 |
| NLE1 | notchless homolog 1 (Drosophila) | 0.256 | -0.062 | 5.76E-05 | 0.032 |
| ERBB2 | erb-b2 receptor tyrosine kinase 2 | 0.245 | -0.464 | 4.70E-05 | 0.029 |
| ACKR2 | atypical chemokine receptor 2 | 0.219 | 0.256 | 2.36E-04 | 0.052 |
| YBEY | ybeY metallopeptidase (putative) | 0.217 | -0.407 | 9.18E-05 | 0.043 |
| ALDOC | aldolase C, fructose-bisphosphate | 0.216 | 0.007 | 2.53E-05 | 0.023 |
| MIR4646 | microRNA 4646 | 0.205 | 0.058 | 4.68E-06 | 0.006 |
| MTUS1 | microtubule associated tumor suppressor 1 | 0.195 | 0.298 | 3.50E-06 | 0.005 |
| MUC12 | mucin 12, cell surface associated | 0.194 | 0.925 | 3.16E-07 | 0.001 |
| NDUFA6-AS1 | NDUFA6 antisense RNA 1 (head to head) | 0.184 | 1.098 | 6.65E-05 | 0.035 |
| ZNF382 | zinc finger protein 382 | 0.177 | -0.130 | 3.19E-06 | 0.005 |
| MIR8061 | microRNA 8061 | 0.162 | 0.023 | 2.07E-06 | 0.004 |
| BTNL3 | butyrophilin-like 3 | 0.161 | 1.818 | 1.88E-07 | 0.001 |
| C6orf10 | chromosome 6 open reading frame 10 | 0.142 | 0.508 | 8.67E-08 | 0.001 |

**Supplemental Table 1** lists mRNAs negatively associated with maternal GDM (P<0.05; FDR<0.1) in the screening group. A paired analysis was conducted and adjusted for maternal weight gain and pregnancy week. Fold-changes (FC) are shown in the 3rd and logarithmic counts per million (logCPM), indicating the relative abundance of the transcript, are shown in the 4th column of the table. Uncorrected P-values and false discovery rates (FDR) are indicated in the 5^th^ and 6^th^ column.

**Supplemental Table 2:** Target mRNAs of miRNA-340 selected for q-PCR validation experiments (sorted by fold-changes)

| Symbol | Name | FC | logCPM | P | FDR |
| --- | --- | --- | --- | --- | --- |
| **GRB10** | growth factor receptor-bound protein 10 | 0.549 | 1.748 | 7.93E-03 | 0.1486 |
| **PAIP1** | poly(A) binding protein interacting protein 1 | 0.509 | 0.726 | 4.33E-02 | 0.1973 |
| CRY2 | cryptochrome circadian clock 2 | 0.559 | 1.904 | 1.22E-02 | 0.1614 |
| ID2 | inhibitor of DNA binding 2, dominant negative helix-loop-helix protein | 0.544 | 2.518 | 9.49E-03 | 0.1547 |
| PITPNB | phosphatidylinositol transfer protein, beta | 0.594 | 3.656 | 1.09E-02 | 0.1582 |
| SPRY3 | sprouty homolog 3 (Drosophila) | 0.644 | 2.767 | 2.50E-02 | 0.1789 |
| SCARB2 | scavenger receptor class B, member 2 | 0.613 | 3.027 | 3.92E-02 | 0.1941 |

**Supplemental Table 2** lists predicted target mRNAs of miRNA-340 which were shown to be negatively associated with maternal GDM (P<0.05) in the screening group in a paired analysis. Fold-changes (FC) are shown in the 3rd and logarithmic counts per million (logCPM) are shown in the 4th column of the table. Uncorrected P-values and false discovery rates (FDR) are indicated in the 5^th^ and 6^th^ column.

**Supplemental Table 3:** mRNAs related with maternal BMI in whole blood cells during pregnancy (sorted by fold-changes)

| Symbol | Name | FC | logCPM | P | FDR |
| --- | --- | --- | --- | --- | --- |
| C6orf10 | chromosome 6 open reading frame 10 | 0.760 | 0.521 | 1.11E-05 | 0.021 |
| NCR1 | natural cytotoxicity triggering receptor 1 | 0.823 | 1.271 | 1.18E-06 | 0.003 |
| RMI2 | RecQ mediated genome instability 2 | 0.842 | -0.497 | 1.57E-04 | 0.083 |
| LOC727751 | golgin A2 pseudogene | 0.842 | -0.069 | 9.65E-05 | 0.070 |
| CDKN1C | cyclin-dependent kinase inhibitor 1C (p57. Kip2) | 0.848 | -0.321 | 1.16E-04 | 0.070 |
| UBE2E2 | ubiquitin-conjugating enzyme E2E 2 | 0.849 | -0.140 | 1.16E-04 | 0.070 |
| RPH3A | rabphilin 3A | 0.858 | 3.037 | 1.55E-06 | 0.003 |
| HIST2H4B | histone cluster 2. H4b | 0.862 | 0.282 | 1.23E-04 | 0.070 |
| HIST2H4A | histone cluster 2. H4a | 0.862 | 0.282 | 1.23E-04 | 0.070 |
| QDPR | quinoid dihydropteridine reductase | 0.872 | -0.405 | 2.00E-04 | 0.097 |
| NCAPG2 | non-SMC condensin II complex. subunit G2 | 0.873 | 0.445 | 6.02E-05 | 0.067 |
| ZNF486 | zinc finger protein 486 | 0.877 | 2.150 | 1.01E-04 | 0.070 |
| S100P | S100 calcium binding protein P | 0.877 | 1.854 | 6.12E-05 | 0.067 |
| DEFA1B | defensin. alpha 1B | 0.890 | 9.338 | 8.70E-05 | 0.070 |
| DEFA1 | defensin. alpha 1 | 0.890 | 9.337 | 8.66E-05 | 0.070 |
| NOL12 | nucleolar protein 12 | 1.083 | 2.110 | 6.96E-05 | 0.068 |
| PEX14 | peroxisomal biogenesis factor 14 | 1.092 | 0.735 | 1.92E-04 | 0.097 |
| BTBD6 | BTB (POZ) domain containing 6 | 1.093 | 1.727 | 2.09E-04 | 0.098 |
| PGAP3 | post-GPI attachment to proteins 3 | 1.108 | 0.971 | 2.21E-05 | 0.037 |
| ITGB3 | integrin. beta 3 (platelet glycoprotein IIIa. antigen CD61) | 1.117 | 5.310 | 6.51E-05 | 0.067 |
| ZNF775 | zinc finger protein 775 | 1.135 | 0.350 | 5.07E-05 | 0.068 |
| CCL3L1 | chemokine (C-C motif) ligand 3-like 1 | 1.136 | 2.183 | 1.22E-08 | 0.001 |
| DDX11L10 | DEAD/H (Asp-Glu-Ala-Asp/His) box helicase 11 like 10 | 1.142 | 1.913 | 1.27E-04 | 0.071 |
| CCL3L3 | chemokine (C-C motif) ligand 3-like 3 | 1.150 | 2.052 | 1.27E-09 | 0.001 |
| SLC12A1 | solute carrier family 12 (sodium/potassium/chloride transporter). member 1 | 1.289 | 3.886 | 1.83E-07 | 0.001 |

**Supplemental Table 3** lists mRNAs related associated with BMI (P<0.05; FDR<0.1) within the screening population. An unpaired analysis was conducted and adjusted for gestational diabetes, age, maternal weight gain, and pregnancy week. Fold changes (FC) are shown in the 3^rd^ and logarithmic counts per million (logCPM), indicating the relative abundance of the transcript, are shown in the 4^th^ column of the table. Uncorrected P-values and false discovery rate as measures of significance of group differences are indicated in the 5^th^ and 6^th^ column.
